# Copeptin: a novel prognostic biomarker in trauma: a review article

**DOI:** 10.1186/s41043-023-00468-1

**Published:** 2023-11-20

**Authors:** Artin Sarkarinejad, Shahram Paydar, Arezou Khosrojerdi, Maryam Hosseini

**Affiliations:** 1 Truama Research Center, Shahid Rajaee (Emtiaz) Trauma Hospital, Shiraz University of Medical Sciences, Shiraz, Iran; 2 Infectious Diseases Research Center, Birjand University of Medical Sciences, Birjand, Iran

**Keywords:** Copeptin, Multiple traumas, Hemorrhagic shock, TBI, Vasopressin

## Abstract

**Background:**

Trauma has a significant impact on the overall health of individuals worldwide, being a leading cause of morbidity and mortality with long-lasting effects. The identification of suitable biomarkers is crucial to predict patient outcomes, providing information about the severity of a condition or the probability of a specific outcome. Hence, in this study, we addressed a new biomarker, copeptin, and discussed its prognostic roles in various trauma researches.

**Main body:**

Copeptin is a peptide derived from the precursor of the hormone vasopressin, which is released in response to stress. Copeptin can serve as a valuable biomarker for determining the severity, prognosis, and outcome of trauma patients. Elevated levels of copeptin are associated with increased mortality and poor clinical outcomes in patients with severe injuries or bleeding. Implementing copeptin measurements in clinical practice can enable healthcare providers to more accurately gauge the degree of trauma and predict patient mortality and morbidity outcomes facilitating prompt interventions and personalized treatment.

**Conclusion:**

The measurement of novel biomarker copeptin can serve as a prognostic molecule for further outcomes in trauma patients. Nevertheless, supplementary research is needed to fully comprehend its role in the development and progression of traumatic injuries.

## Introduction

Trauma is a major cause of morbidity and mortality worldwide, resulting from various sources including motor vehicle accidents, falls, interpersonal violence, and sports-related injuries [1]. According to the World Health Organization, injuries are responsible for over 5 million deaths each year, with trauma being a significant contributor [2]. One of the biggest challenges in managing trauma is the unpredictability of patient outcomes.

Identifying early markers of poor outcomes can help clinicians optimize treatment strategies and improve patient outcomes [3]. Biomarkers are valuable tools for predicting patient outcomes and can guide treatment decisions. However, finding a biomarker that is simple, accessible, and cost-effective is a matter of debate [3]. The ideal biomarker should identify high-risk patients for worse outcomes and predict patient outcomes (mortality and morbidity) with a high degree of accuracy [3]. Additionally, the biomarker should be easy to measure, and the results should be available quickly [4].

Measuring the levels of hormones released by the body during stress, such as corticotropin-releasing hormone (CRH) and arginine vasopressin (AVP), can provide medical experts with insights into a patient's stress response and general state of health. However, measuring the levels of these hormones in circulation is challenging due to their pulsatile secretion, instability, particularly at ambient temperature and rapid clearance from the plasma [5].

Copeptin, a peptide derived from the precursor of vasopressin, releasing in an equal ratio to AVP shows great promise as a biomarker for predicting outcomes in trauma patients [6]. Unlike AVP, copeptin is more stable in circulation and can be easily determined [5]. Studies have found that copeptin levels are elevated in patients suffering from various types of traumas, such as severe traumatic brain injury (TBI) and multiple traumas [7]. This suggests copeptin a valuable biomarker in trauma care, as it can assist medical professionals in assessing the extent of the injury and predicting patient outcomes.

This review study thoroughly explores the correlation between copeptin and its potential applications in the diagnosis, prognosis, and prediction of outcomes related to traumatic events. By evaluating the potential of copeptin as a predictor of clinical outcomes, this review sheds light on a promising avenue for medical research and its implications for patient care.

### Copeptin and the mechanism of its action

Copeptin, a polypeptide discovered by Holwerda et al. in 1972, is a small molecule with a molecular weight of 5 kDa and contains a leucine-rich sequence. It is produced from the same precursor molecule as vasopressin and neurophysin II [8]. When the body experiences stress through osmotic, hemodynamic, and non-specific triggers or in the case of high levels of AVP concentration in the blood, the posterior pituitary gland secretes copeptin [8]. Copeptin plays a critical role in regulating blood pressure and water balance, making it an essential hormone for human health [9]. Although the exact mechanism of copeptin action is not fully comprehended [10]. It is believed that it interacts with the same receptors as AVP, namely the V1a and V2 receptors [11]. This interaction leads to vasoconstriction and water retention, which help sustain blood volume and pressure during stressful times [11].

Copeptin can be easily measured with a sandwich immunoassay and serves as an indirect indicator of AVP levels in circulation [12]. Elevated levels of copeptin may serve as a useful prognostic tool for predicting the onset of various medical conditions such as sepsis, stroke, polycystic kidney disease, and heart failure [13–15]. Additionally, it can help differentiate primary polydipsia from hypovolemic hyponatremia. Furthermore, it has been found to be effective in ruling out acute myocardial infarction in its early stages [16, 17].

### Copeptin and multiple traumas

Multiple traumas refer to the occurrence of two or more injuries to the body, either concurrently or within a short time frame. Patients with multiple traumas require immediate attention and intensive care due to the severity and complexity of their injuries [18]. The presence of multiple injuries increases the risk of complications such as hemorrhagic shock, secondary infections, and organ failure. As a result, healthcare providers face a significant challenge in managing multiple trauma patients, requiring a multidisciplinary approach to ensure the best possible outcomes [18].

Effective care for multiple trauma patients involves rapid identification of injuries and prompt resuscitation to stabilize the patient's condition. A thorough assessment and evaluation are necessary to ascertain the extent and seriousness of the injuries. Treatment options depend on the nature and site of the injuries may include surgical intervention, blood transfusion, and medication administration. Timely intervention and appropriate care are crucial to prevent further injuries and minimize the risk of complications.

Recent studies have highlighted the usefulness of biomarkers such as copeptin and AVP in detecting patients who are vulnerable to hemorrhagic shock and other complications. These biomarkers can offer early warning signs of injury severity and assist in selecting the most appropriate treatment alternatives. A study conducted by Westermann et al. showed the levels of endogenous AVP in patients with multiple traumas were considerably higher than in controls. However, AVP levels decreased within 24 h of ED admission [18]. The results showed that AVP plays an important role in the neuroendocrine response to severe injury in trauma patients. Meanwhile, there was no correlation between AVP and survival in trauma [18]. According to a study conducted by Ipekci et al., patients with multiple traumas and an Injury Severity Score (ISS) greater than 15 had significantly higher levels of blood copeptin on emergency department admission than control group [19]. In addition, non-survivors had higher levels of copeptin, a higher mean ISS and New Injury Severity Score (NISS), and lower Glasgow Coma Scale (GCS) scores than survivors [19]. The study also found a positive correlation between copeptin levels on admission and blood lactate levels, ISS, and NISS, while a negative correlation was found between copeptin levels and GCS. However, the levels of copeptin in patients with head trauma were found to be significantly higher than those without head trauma [19]. These findings suggest that copeptin may be useful in determining the severity, prognosis, and outcome of injury in patients with multiple traumas. Other studies has also demonstrated that mortality rate of patients with severe head trauma was significantly high and determined an inverse correlation between the GCS and mortality [20].

Another study conducted by YC Hsein et al. showed that patients who passed away within 30 days or were admitted to intensive care units exhibited a significant increase in copeptin levels [21]. By integrating copeptin levels with two established trauma severity scores, the trauma-related ISS and the Modified Glasgow Admission Prognostic, researchers were able to more precisely identify adverse outcomes of trauma in patients [21]. This exciting finding may enable medical professionals to detect potential risks earlier and provide more effective treatments for those who are at high risk [21].

In a study by Fulvio Salvo et al., copeptin was found to outperform lactate in identifying major trauma in a mixed group of trauma patients with ISS scores higher than 15 [4]. The study revealed that on-arrival levels of copeptin were more reliable than lactate in identifying patients with such ISS, predicting hospital admission, and determining blood transfusion requirements [4]. These finding suggest that copeptin could be a valuable biomarker for managing trauma patients [4]. However, a separate study showed no significant difference in serum copeptin levels between patients with multiple blunt trauma and the control group during the first 24 h following their injury [22]. Therefore, further research is necessary to confirm these results.

### Copeptin and hemorrhagic shock

Hemorrhagic shock is a serious medical condition that occurs due to significant blood loss, often caused by injury or trauma. It can lead to reduced oxygen and nutrient supply to the body's organs and tissues, which can be life-threatening. Common symptoms of hemorrhagic shock include rapid heartbeat, breathing difficulties, confusion, reduced urine output, and anxiety [23]. If left untreated, the condition can cause organ failure and even death. Treatment usually involves administrating IV fluids, blood transfusions, and medication to improve blood pressure and flow. In some cases, surgery may be necessary to address the root of the bleeding [24].

As previously noted, AVP plays a crucial role in maintaining vasomotor tone, and its depletion can lead to irreversible shock. Sims et al. found elevated AVP and copeptin levels in hypotensive (with a systolic blood pressure less than 90 mmHg) or potentially bleeding trauma patients upon admission, which decreased over time [25]. The study also identified a positive correlation between AVP and copeptin levels and admission to the intensive care unit. These findings suggest that AVP and copeptin could serve as useful biomarkers for identifying at-risk patients and monitoring their response to treatment [25]. Moreover, the study found that initial AVP and copeptin levels were predictive of the need for a blood product transfusion of 10 units or more. Trauma patients frequently experience a relative deficiency of AVP, which subsequently increases their requirement for blood product transfusion [25], suggesting initial copeptin and AVP levels could serve as useful predictors for massive transfusions in trauma patients [25]. Copeptin has the potential to be a valuable clinical biomarker for diagnosing hemorrhagic shock [25]. Yang et al. also found that plasma copeptin levels evaluated upon admission were independent predictors of both progressive hemorrhagic injury and acute traumatic coagulopathy within 6 h of injury [26].

### Copeptin and traumatic brain injury (TBI)

Traumatic brain injury (TBI) can occur as a result of an external force that damages the brain such as falls, car accidents, or blows to the head, causing the brain to move rapidly within the skull. TBI can cause physical, cognitive, and emotional symptoms ranging from mild to severe [27]. Mild TBI, like a concussion, can cause temporary symptoms such as headache, dizziness, and confusion [28]. Moderate-to-severe TBI can lead to more significant symptoms such as loss of consciousness, persistent headaches, seizures, and cognitive deficits. Severe TBI can cause long-term disability, including a coma, vegetative state, or death [28]. Diagnosis of TBI requires a thorough medical evaluation, including imaging tests like computerized tomography (CT) or magnetic resonance imaging (MRI). Treatment for TBI varies depending on the severity of the injury and may involve medication, surgery, and rehabilitative therapy to help the patient regain function and independence [28]. Recent research suggests that copeptin levels can assist in the identification of TBI.

According to a study by Dong et al., patients with TBI had significantly higher plasma copeptin levels immediately after the injury [29]. The levels peaked at 24 h and remained elevated for the next seven days compared to healthy individuals. The study also found a positive correlation between copeptin levels and the risk of 1-month mortality in TBI patients, as well as a negative correlation with the GCS [29]. These findings suggest that copeptin levels may serve as a valuable biomarker for assessing the severity of TBI and predicting patient outcomes. Based on the study, a plasma copeptin cutoff level of 451.8 pg/mL has been established as a reliable predictor of 1-month mortality in TBI patients. Elevated plasma copeptin levels could be considered a potential marker to predict patient outcomes (mortality and morbidity) [29].

A recent meta-analysis was conducted to assess the effectiveness of plasma copeptin levels in diagnosing and predicting outcomes in patients with TBI [30]. The analysis revealed that elevated plasma copeptin levels were significantly associated with TBI, mortality, and poor outcomes [30]. In a comparison study by Zhang et al., plasma concentrations of several biomarkers were found to be significantly higher in severe TBI patients compared to healthy controls and non-survivors compared to survivors. However, only plasma copeptin concentration was found to significantly improve the prognostic value of the GCS score [31]. As a result, the study suggests that plasma copeptin concentration could be a valuable tool in TBI diagnosis and prognostication, aiding in predicting long-term clinical outcomes following severe TBI [31]. According to a different meta-analysis by Choi et al., patients who passed away due to TBI had significantly higher initial plasma copeptin levels than those who survived. Additionally, patients with unfavorable outcomes had considerably higher copeptin levels compared to those with favorable outcomes. These findings highlight the potential utility of plasma copeptin levels for predicting mortality and unfavorable neurological outcomes [32].

Cavus et al. conducted a study and found although there was no significant difference in copeptin levels between TBI patients and the control group upon admission and 6 h after trauma, patients who passed away within a month of TBI had higher Δ-Copeptin levels, indicating a difference between the copeptin level at the 6th hour after trauma and that at admission [33]. Patients with no improvement in the modified Rankin score also had higher Δ-copeptin levels compared to those who showed improvement [33]. The study identified a Δ-copeptin level of 0.51 ng/mL as the best cutoff point for predicting mortality, while a level of 0.23 ng/mL was identified as the best cutoff point for predicting improvement in the modified Rankin score. Therefore, plasma Δ-copeptin levels could be a useful tool in predicting the prognosis of TBI patients [33].

After experiencing a TBI, individuals may face various health complications that can be broadly categorized into two types: physical and mental diseases [34]. Physical complications may include seizures, chronic pain syndromes, balance problems, and sleep disorders such as insomnia or sleep apnea. These complications can last for weeks, months, or even years [34]. Mental diseases that can develop after TBI include depression, anxiety, post-traumatic stress disorder, chronic traumatic encephalopathy, and cognitive impairment [34]. Although not everyone who experiences TBI will develop a disease, it is important to seek medical attention for any physical or mental symptoms. Researchers have also studied the role of copeptin in predicting the development of diseases that may follow a TBI. Cardoso et al. investigated the biomarkers of impulsivity in mild TBI (mTBI) and found that serum levels of copeptin had a correlation with impulsivity in individuals with mTBI [35]. This highlights the potential value of copeptin as a biomarker that not only predicting outcomes but also identifies potential health concerns that may arise following a TBI.

### Copeptin and pediatric trauma

Pediatric trauma refers to physical injuries experienced by individuals under the age of 18 years, with head injuries, fractures, and soft tissue injuries being the most typical forms [36]. Managing pediatric trauma is complex and requires a multidisciplinary approach involving specialists, surgeons, and other healthcare professionals [36].

Studies by Chao Lin et al. have confirmed the significance of copeptin in cases of pediatric TBI [37]. It was found that plasma copeptin levels were higher in TBI children than in healthy children [37] and were identified as an independent predictor of 6-month mortality and long-term clinical outcomes [37]. Additionally, a study investigating the value of serum copeptin and S100B protein in combination with uric acid in the prognosis of children with TBI showed that children with more severe TBI had higher levels of copeptin, S100B protein, and uric acid levels. These molecules were also associated with the prognosis of TBI, specially on the third day of admission and had a stronger predictive value for mortality [38]. Furthermore Baumann et al. explored the role of copeptin in arterial hypotension and its association with severity of disease in critically ill children. They showed that copeptin levels were notably higher in critically ill children compared to the levels typically observed in healthy children. They also showed a positive correlation between copeptin and clinical indicators that reflect the severity of the disease, suggesting its potential as a marker for predicting short-term outcomes [39].

### Copeptin: the superior marker for clinical assessment

Copeptin, a stable biomarker, has several advantages over other markers like base excesses or lactate in trauma conditions. Here are a few key advantages:

- *Early detection*: Copeptin levels rise rapidly in response to stress [40, 41], making it useful for the early diagnosis and monitoring of trauma conditions. This can potentially allow for prompt medical interventions.
- *Stability*: Copeptin is a stable peptide, meaning it does not degrade easily and can be reliably measured in blood samples even if there are delays in transportation or processing [6].
- *Predictive value*: Copeptin has been found to have a higher specificity for detecting trauma-related complications, such as sepsis or acute kidney injury, compared to lactate. This means that copeptin can provide more accurate information about the presence and severity of these conditions [4].
- *Versatility*: Copeptin is easy to measure. It can be measured using various laboratory techniques, including immunoassays and point-of-care testing, whereas measuring lactate levels requires specialized equipment and is more time-consuming [6].

When considering the potential benefits of copeptin, it is crucial to remember that it should not be the sole basis for accurate assessments. It should be used alongside other clinical information. Additionally, copeptin is still under investigation, and its role in clinical practice is constantly evolving. Nevertheless, the early findings regarding its stability, correlation with trauma severity, and predictive abilities make it an intriguing area of research.

## Conclusion

Trauma has a significant impact on global health being a leading cause of morbidity and mortality worldwide. Its effects can be long-lasting making it essential to improve patient care and identify biomarkers that can predict patient outcomes. Although the correlation between copeptin and trauma outcomes is still a topic of ongoing debate, there is growing evidence of its potential as a biomarker for trauma and post-trauma diseases to assist in determining the severity, prognosis, and outcome of trauma patients. Elevated levels of copeptin have been associated with increased mortality, severe injuries, and bleeding. Therefore, the use of copeptin as a biomarker in trauma cases provides promising possibilities for improving patient care and optimizing treatment approaches. However, further research is necessary to fully understand copeptin’s potential as a biomarker in trauma management.

## Data Availability

Not applicable.

## Abbreviations

AVP: Arginine vasopressin
CT: Computerized tomography
CRH: Corticotropin-releasing hormone
ISS: Injury severity score
GCS: Lower Glasgow coma scale
MRI: Magnetic resonance imaging
mTBI: Mild TBI
NISS: New injury severity score
TBI: Traumatic brain injury

## References

[1] Miclau T (2019). Understanding trauma systems: a global need. OTA Int.

[2] Organization World Health. Injuries and violence 2021.

[3] Burke HB (2016). Predicting clinical outcomes using molecular biomarkers. Biomark Cancer.

[4] Salvo F, Luppi F, Lucchesi DM, Canovi S, Franchini S, Polese A (2020). Serum Copeptin levels in the emergency department predict major clinical outcomes in adult trauma patients. BMC Emerg Med.

[5] Lewandowski KC, Lewiński A, Skowrońska-Jóźwiak E, Malicka K, Horzelski W, Brabant G (2017). Copeptin as a marker of an altered CRH axis in pituitary disease. Endocrine.

[6] Morgenthaler NG, Struck J, Alonso C, Bergmann A (2006). Assay for the measurement of copeptin, a stable peptide derived from the precursor of vasopressin. Clin Chem.

[7] Ponte B, Pruijm M, Ackermann D, Vuistiner P, Guessous I, Ehret G (2015). Copeptin is associated with kidney length, renal function, and prevalence of simple cysts in a population-based study. J Am Soc Nephrol.

[8] Christ-Crain M, Fenske W (2016). Copeptin in the diagnosis of vasopressin-dependent disorders of fluid homeostasis. Nat Rev Endocrinol.

[9] Dobša L, Cullen EK (2013). Copeptin and its potential role in diagnosis and prognosis of various diseases. Biochem Medica.

[10] Abdelmageed M, Güzelgül F. Copeptin: up-to-date diagnostic and prognostic role highlight. Anal Biochem. 2023;673:115181. 10.1016/j.ab.2023.115181 37247750

[11] Szczepanska-Sadowska E, Zera T, Sosnowski P, Cudnoch-Jedrzejewska A, Puszko A, Misicka A (2017). Vasopressin and related peptides; potential value in diagnosis, prognosis and treatment of clinical disorders. Curr Drug Metab.

[12] Christ-Crain M (2019). Vasopressin and Copeptin in health and disease. Rev Endocr Metab Disord.

[13] Gille J, Schmidt J, Kremer T, Sablotzki A (2019). Evaluation of MR-proANP and copeptin for sepsis diagnosis after burn injury. J Crit Care.

[14] El Boustany R, Tasevska I, Meijer E, Kieneker LM, Enhörning S, Lefèvre G, et al. Plasma copeptin and chronic kidney disease risk in 3 European cohorts from the general population. JCI Insight. 2018;3(13):e121479. 10.1172/jci.insight.121479 PMC6124520 29997293

[15] Choi K-S, Kim HJ, Chun H-J, Kim JM, Yi H-J, Cheong J-H (2015). Prognostic role of copeptin after stroke: a systematic review and meta-analysis of observational studies. Sci Rep.

[16] Mu D, Ma C, Cheng J, Zou Y, Qiu L, Cheng X (2022). Copeptin in fluid disorders and stress. Clin Chim Acta.

[17] Reinstadler SJ, Klug G, Feistritzer H-J, Metzler B, Mair J. Copeptin testing in acute myocardial infarction: ready for routine use? Disease Markers. 2015;2015:614145. 10.1155/2015/614145 PMC4415476 25960596

[18] Westermann I, Dünser MW, Haas T, Jochberger S, Luckner G, Mayr VD (2007). Endogenous vasopressin and copeptin response in multiple trauma patients. Shock.

[19] Ipekci A, Ozkan S, Ikizceli I, Durukan P, Avçarogullari L, Muhtaroglu S (2013). Correlation between blood copeptin level and blood lactate level, trauma severity scores, and clinical parameters. Int Med J.

[20] Dur A, Cander B, Koçak S, Girişgin AS, Gül M, Koyuncu F (2009). Acil yoğun bakım da çoklu travma hastaları ve skorlama sistemleri. Akademik Acil Tıp Dergisi.

[21] Hsein Y-C, Wu I-J, Tan J, Huang S-S, Lu K-T, Su C-H (2023). Serum levels of copeptin predict adverse outcomes and improve risk prediction of TRISS and MGAP scores in patients with multiple trauma: a single-center prospective cohort study. J Trauma Acute Care Surg.

[22] Ağuş T, Az A, Akdemir T, Söğüt Ö. The utility of serum copeptin levels for the determination of injury severity and prognosis in adult patients with multiple blunt trauma. 2022.

[23] Cannon Jeremy W (2018). Hemorrhagic shock. N Engl J Med.

[24] Gutierrez G, Reines H, Wulf-Gutierrez ME (2004). Clinical review: hemorrhagic shock. Crit Care.

[25] Sims CA, Guan Y, Bergey M, Jaffe R, Holmes-Maguire L, Martin N (2017). Arginine vasopressin, copeptin, and the development of relative AVP deficiency in hemorrhagic shock. Am J Surg.

[26] Yang D-B, Yu W-H, Dong X-Q, Du Q, Shen Y-F, Zhang Z-Y (2014). Plasma copeptin level predicts acute traumatic coagulopathy and progressive hemorrhagic injury after traumatic brain injury. Peptides.

[27] Capizzi A, Woo J, Verduzco-Gutierrez M (2020). Traumatic brain injury: an overview of epidemiology, pathophysiology, and medical management. Med Clin.

[28] Rauchman SH, Albert J, Pinkhasov A, Reiss AB (2022). Mild-to-moderate traumatic brain injury: a review with focus on the visual system. Neurol Int.

[29] Dong X-Q, Huang M, Yang S-B, Yu W-H, Zhang Z-Y (2011). Copeptin is associated with mortality in patients with traumatic brain injury. J Trauma Acute Care Surg.

[30] Zhang J, Wang H, Li Y, Zhang H, Liu X, Zhu L (2021). The diagnosis and prognostic value of plasma copeptin in traumatic brain injury: a systematic review and meta-analysis. Neurol Sci.

[31] Zhang Z-Y, Zhang L-X, Dong X-Q, Yu W-H, Du Q, Yang D-B (2014). Comparison of the performances of copeptin and multiple biomarkers in long-term prognosis of severe traumatic brain injury. Peptides.

[32] Choi K-S, Cho Y, Jang B-H, Kim W, Ahn C, Lim TH (2017). Prognostic role of copeptin after traumatic brain injury: a systematic review and meta-analysis of observational studies. Am J Emerg Med.

[33] Cavus U, Yildirim S, Gurer B, Dibek K, Yilmaz D, Ozturk G (2014). The prognostic value of plasma Δ-copeptin levels in patients with isolated traumatic brain injury. Eur J Trauma Emerg Surg.

[34] Gardner RC, Yaffe K (2015). Epidemiology of mild traumatic brain injury and neurodegenerative disease. Mol Cell Neurosci.

[35] de Freitas Cardoso MG, de Barros JLVM, de Queiroz RAB, Rocha NP, Silver C, da Silva AS (2023). Potential biomarkers of impulsivity in mild traumatic brain injury: a pilot study. Behav Brain Res.

[36] Schafermeyer R (1993). Pediatric trauma. Emerg Med Clin North Am.

[37] Lin C, Wang N, Shen Z-P, Zhao Z-Y (2013). Plasma copeptin concentration and outcome after pediatric traumatic brain injury. Peptides.

[38] Lin L, Mai L, Chen G, Zhao E, Xue M (2020). Value of serum copeptin and S100B protein combined with uric acid in the prognosis of children with traumatic brain injury. Zhonghua Wei Zhong Bing Ji Jiu Yi Xue.

[39] Baumann P, Gotta V, Atkinson A, Deisenberg M, Hersberger M, Roggia A (2022). Copeptin release in arterial hypotension and its association with severity of disease in critically Ill children. Children.

[40] Christ-Crain M (2010). The stress hormone copeptin: a new prognostic biomarker in acute illness. Swiss Med Wkl.

[41] Martino M, Arnaldi G (2021). Copeptin and stress. Endocrines.

